# Risk of lymph-node metastasis in early onset T1 colorectal cancer: a systematic review and meta-analysis

**DOI:** 10.1007/s00535-026-02465-7

**Published:** 2026-06-29

**Authors:** Hiromu Fukuda, Yoshito Hayashi, Shinji Yoneda, Yujiro Adachi, Ayaka Tajiri, Eiji Kimura, Ryotaro Uema, Takeo Yoshihara, Yoshiki Tsujii, Takahiro Kodama, Tetsuo Takehara

**Affiliations:** Department of Gastroenterology and Hepatology, The University of Osaka Graduate School of Medicine, Yamadaoka, 2-2, Suita, Osaka 565-0871 Japan

**Keywords:** Colorectal cancer, Early onset, T1, Lymph-node metastasis

## Abstract

**Background:**

Despite the increasing incidence of early onset T1 colorectal cancer (T1 CRC), its features remain poorly understood. We examined whether early onset T1 CRC is associated with a higher rate of lymph-node metastasis (LNM).

**Methods:**

We included studies reporting age-specific LNM rates for patients aged < 50 and ≥ 50 years. Sensitivity analyses were performed to assess the impact of overlapping and selected subset datasets. Subgroup analyses were conducted to evaluate the association between clinicopathological variables and age. The risk of bias was assessed using the Risk of Bias In Non-randomized Studies of Interventions Version 2.

**Results:**

Of the 1214 records identified, 10 studies comprising 11 databases were included. After excluding overlapping and selected subset data, six databases from five studies with 29,132 patients were analyzed. Of these, 2759 (9.4%) were aged < 50 years at diagnosis, and the pooled LNM rates were 19.2% (95% confidence interval [CI], 17.8–20.6) in early onset and 11.1% (95% CI 10.7–11.5) in late-onset T1 CRC, representing an absolute difference of approximately 8%. Early onset T1 CRC was associated with higher odds of LNM than late-onset disease (OR: 1.94; 95% CI 1.75–2.15; *I*^*2*^ = 0%).

Despite serious confounding in three studies, multivariable analyses adjusting for clinical covariates showed that younger age was associated with higher odds of LNM, and adjusted ORs for older patients ranged between 0.46 and 0.90 compared with those aged < 50 years.

**Conclusions:**

This meta-analysis demonstrated that early onset T1 CRC is associated with higher odds of LNM compared with late-onset T1 CRC in surgically treated patients.

**Supplementary Information:**

The online version contains supplementary material available at https://doi.org/10.1007/s00535-026-02465-7.

## Introduction

Colorectal cancer (CRC) is one of the most prevalent malignancies worldwide [1]. Although most CRCs are diagnosed in old age, approximately 10% occur in individuals under age 50, known as early-onset CRC (EO-CRC). Recent studies have reported that the annual incidence of EO-CRC is increasing by 1.4% [2–4]. Several hypotheses have been proposed to explain the rising incidence, including lifestyle-related factors [5–7]. Clinically, features of EO-CRC differ significantly from those of late-onset CRC (LO-CRC). Previous studies have shown that EO-CRC is often detected at a more advanced stage due to delayed diagnosis as well as its higher biological malignancy [8–12]. These factors may contribute to poorer outcomes in younger patients, despite their favorable baseline health status, indicating the necessity of cancer detection at an earlier stage. Hence, in the coming years, the incidence of early stage CRC, such as T1 CRC, may also increase among young adults as a result of changes in screening algorithms [13–15]. T1 CRC is generally considered a curable disease with a favorable 5-year survival when treated appropriately. However, lymph-node metastasis (LNM) occurs in approximately 10% of patients with T1 CRC [16–18]. Therefore, assessment of LNM risk is crucial for determining management in patients with T1 CRC [19, 20]. Although numerous studies have investigated risk factors for LNM in T1 CRC, age has not typically been considered a common risk factor [21, 22]. The limited number of studies specifically investigating the association between younger age and LNM may account for this gap. Consequently, no consensus has been reached on whether EO-T1 CRC should be managed using the same treatment strategies and surveillance protocols as those for LO-T1 CRC.

In this study, we aimed to compare the LNM rates between EO- and LO-T1 CRC through an integrated analysis of previously published reports.

## Methods

This meta-analysis followed the Meta-Analysis of Observational Studies in Epidemiology (MOOSE) guidelines [23]. The Protocol, including the eligibility criteria, quality assessment, and method of statistical analysis, was registered with the International Prospective Register of Systematic Reviews (PROSPERO registration no. CRD 42025625333).

### Eligibility criteria

We included all studies that analyzed the prevalence of LNM in patients with CRC. Studies were eligible if they reported age-specific LNM rates in patients with T1 CRC. EO-T1 CRC was predefined as cases diagnosed at < 50 years. Although studies using different age cutoffs were identified during the screening process, only studies that clearly stratified patients into two age groups (< 50 and ≥ 50 years) were included. LNM was defined as concurrent LNM at the time of surgery. Regarding treatment, both endoscopic and surgical resection cases were included, and each study was reviewed after screening to determine the treatment used. Studies without age-specific data, unavailable full-text articles, case reports, small series (< 10 patients) with insufficient statistical power, or duplicate publications (to avoid double-counting) were excluded.

### Information source and search strategy

The PubMed/MEDLINE, SCOPUS, Cochrane Library, Web of Science, and ICHUSHI databases were searched to identify published studies. All reference lists of the included papers and relevant reviews were searched, and appropriate papers were considered for inclusion. Abstracts from major conferences were screened, and unpublished studies were considered if sufficient data could be extracted or obtained by contacting the authors. The search strategy was designed according to the “Participants, Interventions, Comparisons, Outcomes and Study design” (PICOS) statement and combined controlled terms such as Medical Subject Headings (MeSH) and free-text keywords. Representative MeSH terms included “colorectal neoplasms,” “neoplasm invasiveness,” “lymphatic metastasis,” and “young adult.” Corresponding free-text keywords included “colorectal cancer,” “T1,” “submucosal invasion,” “lymph-node metastasis,” “LNM,” and “early onset.” The full electronic search strategy for each bibliographic database is provided in the Supplementary Information. The investigators searched for eligible studies published before December 31, 2024.

### Study selection and data extraction

One reviewer conducted a literature search in cooperation with a clinical librarian. Two main reviewers and a third reviewer resolved any disagreements. Duplicate records were removed before title and abstract screening. Two reviewers independently screened all the titles and abstracts for eligibility. The screening process was documented, and a study-specific extraction form was used to collect data. The risk of bias was assessed using The Risk of Bias In Non-randomized Studies of Interventions Version 2 (ROBINS-I V2) [24].

### Statistical analysis

We performed a meta-analysis to evaluate the rate of LNM in EO- and LO-CRC using the inverse variance method. Heterogeneity was measured using Cochran’s *Q* test and the *I*^2^ statistic. A Cochran’s *Q* test with *p* < 0.05 or an *I*^2^ statistic ≥ 50% was considered indicative of substantial heterogeneity. If substantial heterogeneity was present, a random-effects model was used to estimate the pooled effect; otherwise, a fixed-effects model was used when heterogeneity was not detected. All statistical analyses were conducted using Review Manager (version 5.4.1; The Cochrane Collaboration, Copenhagen, Denmark) to combine results across studies. Publication bias was assessed using funnel plots with Egger’s test.

### Primary analysis

As the eligible studies included overlapping and selected subset datasets, we predefined the studies and datasets for inclusion in the primary analysis. Studies with data available only for selected subsets were excluded from the primary analysis and included in sensitivity analyses. Among SEER-based studies, we selected those with non-overlapping study periods to minimize potential bias and avoid duplication and prioritized multiple independent datasets to enhance the robustness of the analysis.

### Sensitivity analysis

As some eligible studies used data from overlapping periods within the same database, such as SEER database, we conducted three sensitivity analyses: (i) excluding all SEER data [25–29]; (ii) including all SEER data [17, 25–33]; and (iii) integrating SEER-based studies [17, 30–33] and analyzing together with the other studies [25–29]. All SEER-based studies were pooled using an inverse variance fixed-effect model to generate a single SEER-based estimate, which was then included as one dataset in the analysis alongside the remaining non-SEER-based studies. In addition, we performed a sensitivity analysis that included subset data restricted to patients at low risk for LNM [27] and assessed the robustness of the findings using alternative effect models.

### Subgroup analysis

Subgroup analyses were performed based on clinicopathological feature data, such as sex, tumor location, size, tumor grade, invasion depth, and lymphovascular invasion status. Although some studies were not included in the primary analysis, they met the eligibility criteria, and the data used were confirmed to be non-duplicative.

Adjusted odds ratios (ORs) from five databases in four studies that performed multivariable logistic regression analyses incorporating age < 50 years as a covariate in LNM models were assessed [17, 26, 28, 33]. As adjusted ORs were derived using different age categorizations across studies, a multivariable meta-analysis was not conducted, and the analysis was limited to a qualitative literature review.

## Results

### Study selection

A total of 1214 articles were identified. After the first screening, 71 full-text articles available references were included in the secondary screening. After excluding 61 that did not meet the eligibility criteria, 10 articles reporting age-specific LNM rates in patients with T1 CRC were extracted [17, 25–33]. The reasons for exclusion were as follows: (i) use of age cutoffs other than 50 years (*n* = 12); (ii) both age- and T stage-specific LNM rates unavailable (*n* = 13); (iii) T stage-specific LNM rates unavailable (*n* = 26); (iv) age-specific LNM rates unavailable (*n* = 5); and (v) results for only a single age group reported (*n* = 5). None of the conference abstracts and unpublished data were available.

**Fig. 1:**
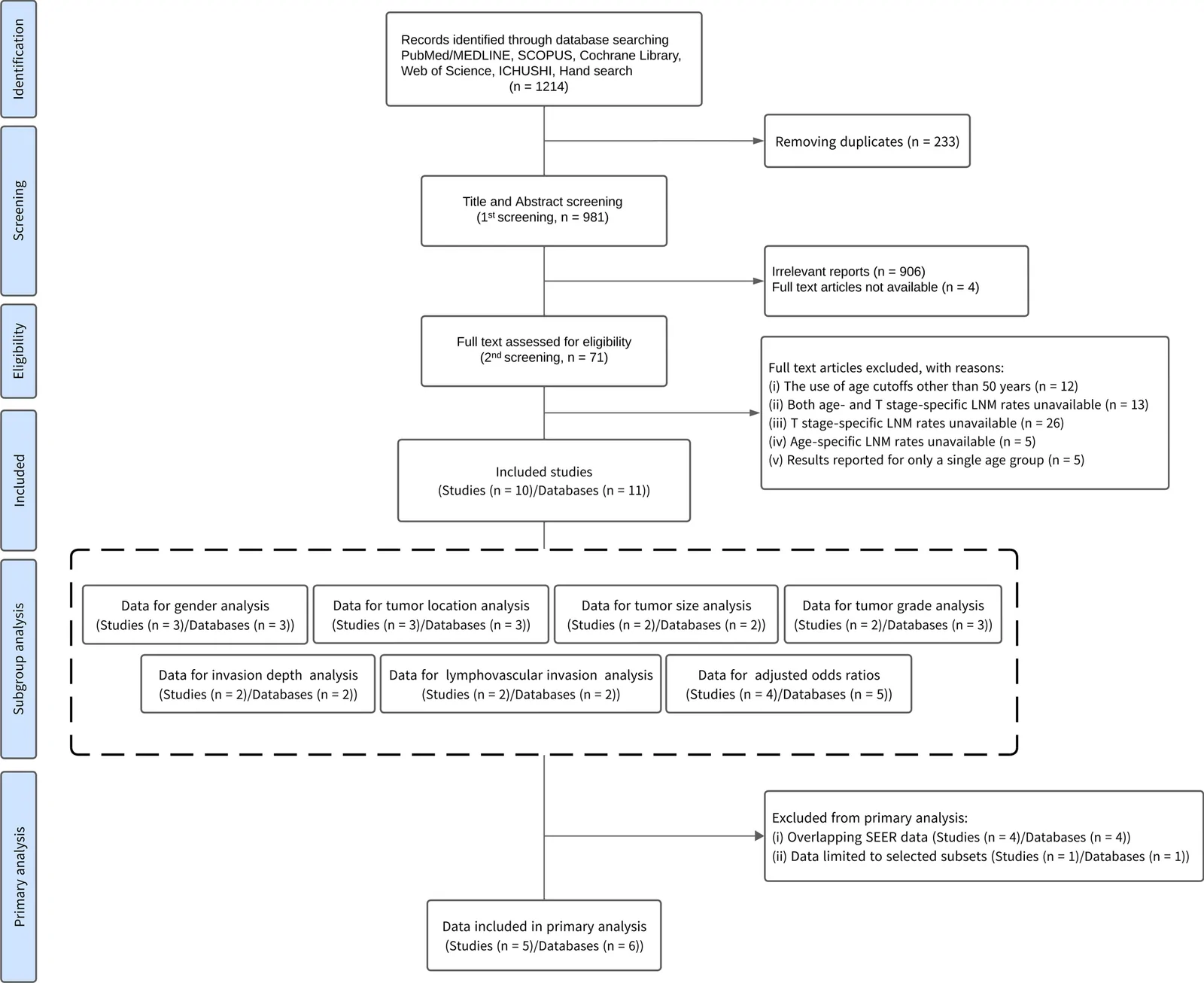
The flow diagram of the study selection process

Of these 10 studies [17, 25–33], one reported by Tang et al. [28] used two available databases within a single article. Because the data were completely independent, the two databases were included separately. Finally, 11 databases from 10 studies were included in the meta-analysis (Fig. 1) [17, 25–33]. All references were published within 10 years from 2016 to 2024; six studies were case control studies, and four were retrospective cohort studies. Three of these were data from a single institute [25, 28, 29], and eight were registry databases [17, 26–29, 31–33]. EO-CRC was defined as CRC if the patient was < 50 years of age at diagnosis. Ultimately, all included studies consisted of patients who underwent surgical resection with lymph-node dissection. The outcome was the number of LNM, and the OR comparing the rate of LNM in EO-T1 CRC to that in LO-T1 CRC ranged from 1.24 to 2.71. LNM was evaluated as concurrent LNM at the time of surgery in all studies [17, 25–33]. Cases with unknown N stage were excluded in eight studies [17, 26–28, 30–33], and six studies included only patients with examined LNs [17, 26, 27, 31–33]. Radical surgery with LN dissection was performed in eight studies [17, 25–27, 29, 31–33]. Additionally, nine studies excluded patients who received neoadjuvant therapy prior to surgery [25–33]. Only one study reported endoscopic resection as a prior treatment [26], and two studies described patients as having undergone surgery with or without prior endoscopic resection [27, 29] (Table 1).

**Table 1:** Characteristics of included studies

Author (year)	Study design	Country (author)	Database	Year (database)	Country (database)	Purpose of study	Definition of EO-CRC	Definition of LNM	Definition of treatment
Wang 2024 [29]	Case–control study	China	The Sixth Affiliated Hospital, Sun Yat-sen University	2010–2021	China	To develop a practical prediction model for LNM in T1 CRC	< 50	Concurrent LNM at the time of surgery	Radical resection
Rönnow 2022 [27]	Retrospective cohort study	Sweden	SCRCR	2009–2017	Sweden	To identify clinical risk factors of LNM in T1 CRC	< 50	Concurrent LNM at the time of surgery	Radical resection with lymph node dissection
Naffouje 2022 [26]	Case–control study	USA	NCDB	2004–2018	USA	To construct a clinical tool for LNM risk estimation of endoscopically excised CRC	< 50	Concurrent LNM at the time of surgery	Radical resection with lymph-node dissection
Tang 2021 hospital data [28]	Retrospective cohort study	China	Hospital of Nanchang University	2011–2019	China	To clarify differences in LNM and prognosis between early onset and late-onset CRC	< 50	Concurrent LNM at the time of surgery	Surgical resection
Tang 2021 SEER data [28]	Retrospective cohort study	China	SEER	2010–2015	USA	To clarify differences in LNM and prognosis between early onset and late-onset CRC	< 50	Concurrent LNM at the time of surgery	Surgical resection
Ahn 2021 [30]	Case–control study	Korea	SEER	2004–2016	USA	To develop a practical prediction model for LNM in early CRC	< 50	Concurrent LNM at the time of surgery	Surgical resection
Zhang 2020 [33]	Retrospective cohort study	China	SEER	1988–2015	USA	To investigate the association between age of CRC onset and LNM	< 50	Concurrent LNM at the time of surgery	Radical resection with lymph node dissection
Guo 2020 [17]	Case–control study	China	SEER	2004–2016	USA	To find clinicopathological factors associated with LNM in T1 CRC	< 50	Concurrent LNM at the time of surgery	Radical resection with lymph-node dissection
Xie 2019 [32]	Case–control study	China	SEER	2004–2014	USA	To quantify the effect of younger age on LNM in CRC with mucosal invasion	< 50	Concurrent LNM at the time of surgery	Radical resection with lymph-node dissection
Aytac 2016 [25]	Case–control study	USA	Cleveland Clinic	1997–2014	USA	To evaluate the impact of tumor location on LNM in T1 CRC	< 50	Concurrent LNM at the time of surgery	Radical resection
Meyer 2016 [31]	Retrospective cohort study	USA	SEER	1988–2008	USA	To quantify the effect of younger age on LNM in rectal cancer	< 50	Concurrent LNM at the time of surgery	Radical resection with lymph-node dissection

Author (year)	Prior treatment	Exclusion criteria regarding LNM and treatment	Outcome	Events (EO-CRC)	Sample (EO-CRC)	Events (LO-CRC)	Sample (LO-CRC)	OR	95% CI
Wang 2024 [29]	With or without endoscopic resection	Neoadjuvant radiotherapy and chemotherapy	LNM	13	111	45	466	1.24	0.64–2.39
Rönnow 2022 [27]	With or without endoscopic resection	Missing data on analyzed lymph nodes, neoadjuvant treatment	LNM	8	48	69	1005	2.71	1.22–6.02
Naffouje 2022 [26]	Endoscopic resection	Inadequate nodal sampling during surgical resection, defined as < 12 nodes in the final specimen, neoadjuvant chemotherapy or radiation	LNM	114	587	566	5482	2.09	1.68–2.61
Tang 2021 hospital data [28]	Not stated	No recorded information about N stage, chemotherapy before surgery	LNM	19	119	32	357	1.93	1.05–3.55
Tang 2021 SEER data [28]	Not stated	No recorded information about N stage, chemotherapy before surgery	LNM	218	1040	1433	12,044	1.96	1.67–2.30
Ahn 2021 [30]	Not stated	Insufficient N stage data, neoadjuvant radiotherapy	LNM	314	2242	2229	24,491	1.63	1.43–1.85
Zhang 2020 [33]	Not stated	The number of retrieved lymph nodes were not available, preoperative radiotherapy or chemotherapy	LNM	426	2675	2475	26,726	1.86	1.66–2.07
Guo 2020 [17]	Not stated	Regional lymph nodes examined < 12, Nx or Mx	LNM	355	1885	1936	15,424	1.62	1.43–1.83
Xie 2019 [32]	Not stated	No lymph node resected, radiotherapy prior to surgery	LNM	721	3048	5637	38,442	1.8	1.65–1.97
Aytac 2016 [25]	Not stated	Preoperative chemoradiation	LNM	5	28	19	204	2.12	0.72–6.21
Meyer 2016 [31]	Not stated	Local excision or local destruction procedures, radiotherapy prior to surgery	LNM	158	874	831	7820	1.86	1.54–2.24

*EO-CRC* early onset colorectal cancer, *LO-CRC* late-onset colorectal cancer, *CRC* colorectal cancer, *LNM* lymph-node metastasis, *OR* odds ratio, *CI* confidence interval, *SCRCR* Swedish Colorectal Cancer Registry, *NCDB* National Cancer Database, *SEER* Surveillance Epidemiology and End Results

### Risk of bias

We assessed the risk of bias using ROBINS-I, version 2. The risk of deviation from intended interventions was judged to be low in all studies, because age was already definitive at the time of the study, with no missing data. However, the risk of confounding bias was judged to be serious in three studies, because the analyses including other potential confounding factors were not performed [25, 30, 31]. The assessment outcomes for each domain of bias and overall risk of bias for each study are summarized (Fig. S1). Given that several domains were found to carry a high risk of bias, we concluded that the overall risk of bias was serious across all three studies [25, 30, 31].

### LNM in EO- versus LO-T1 CRC

Among the 10 eligible studies, a total of 11 databases were identified, including five non-SEER and six SEER-based datasets. To minimize potential bias and avoid duplication, four overlapping SEER-based datasets were excluded from the primary analysis [17, 30, 32, 33]. In addition, one non-overlapping study [27] was excluded, because the available data were restricted to a low-risk subgroup without established risk factors for LNM. Consequently, the primary analysis incorporated data from six databases reported in five studies, covering a total of 29,132 patients with T1 CRC [25, 26, 28, 29, 31]. Of these, 2,759 (9.4%) were aged < 50 years at the time of diagnosis. The pooled LNM rates in EO- and LO-T1 CRC were 19.2% (95% confidence interval [CI], 17.8–20.6) and 11.1% (95% CI, 10.7–11.5), respectively.

**Fig. 2:**
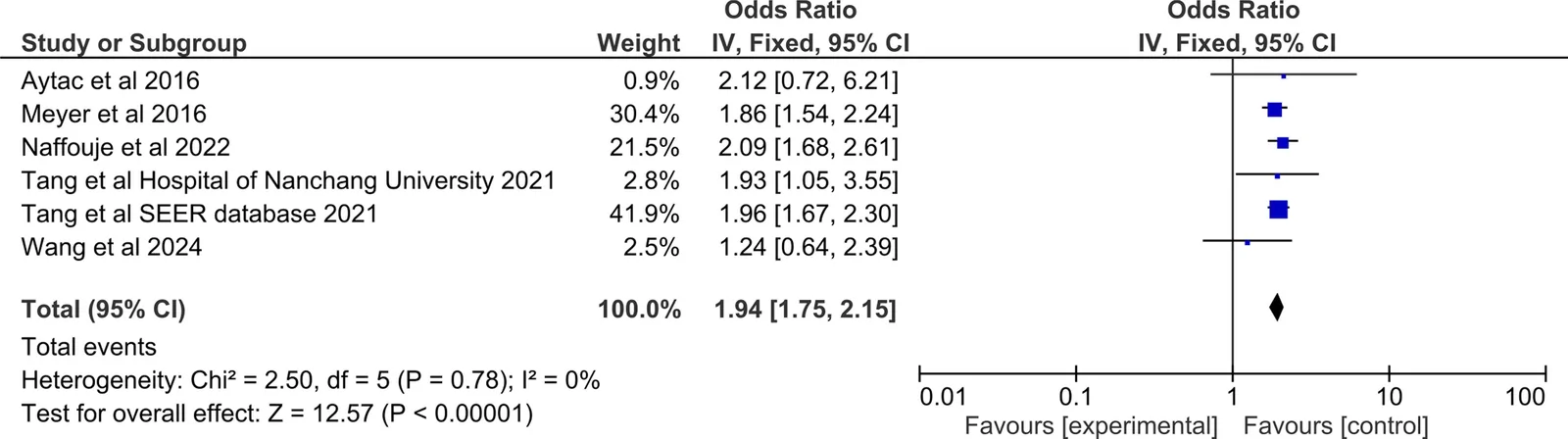
Forest plots for each outcome comparing the odds ratios of LNM between early onset and late-onset T1 CRC in a fixed-effects model using the inverse variance method. *LNM* lymph-node metastasis, *CRC* colorectal cancer

In this meta-analysis, the number of LNM in patients with EO-T1 CRC was significantly higher than that in the LO group (a pooled OR: 1.94; 95% CI, 1.75–2.15, *p* < 0.05 in the fixed-effect model; Fig. 2). The LNM rate was higher in the EO-T1 CRC group in all studies, and we applied a fixed-effects model, because no heterogeneity was observed (*χ*^2^ = 2.50, df = 5, *p* = 0.78, and *I*^2^ = 0%).

### Sensitivity analysis

**Fig. 3:**
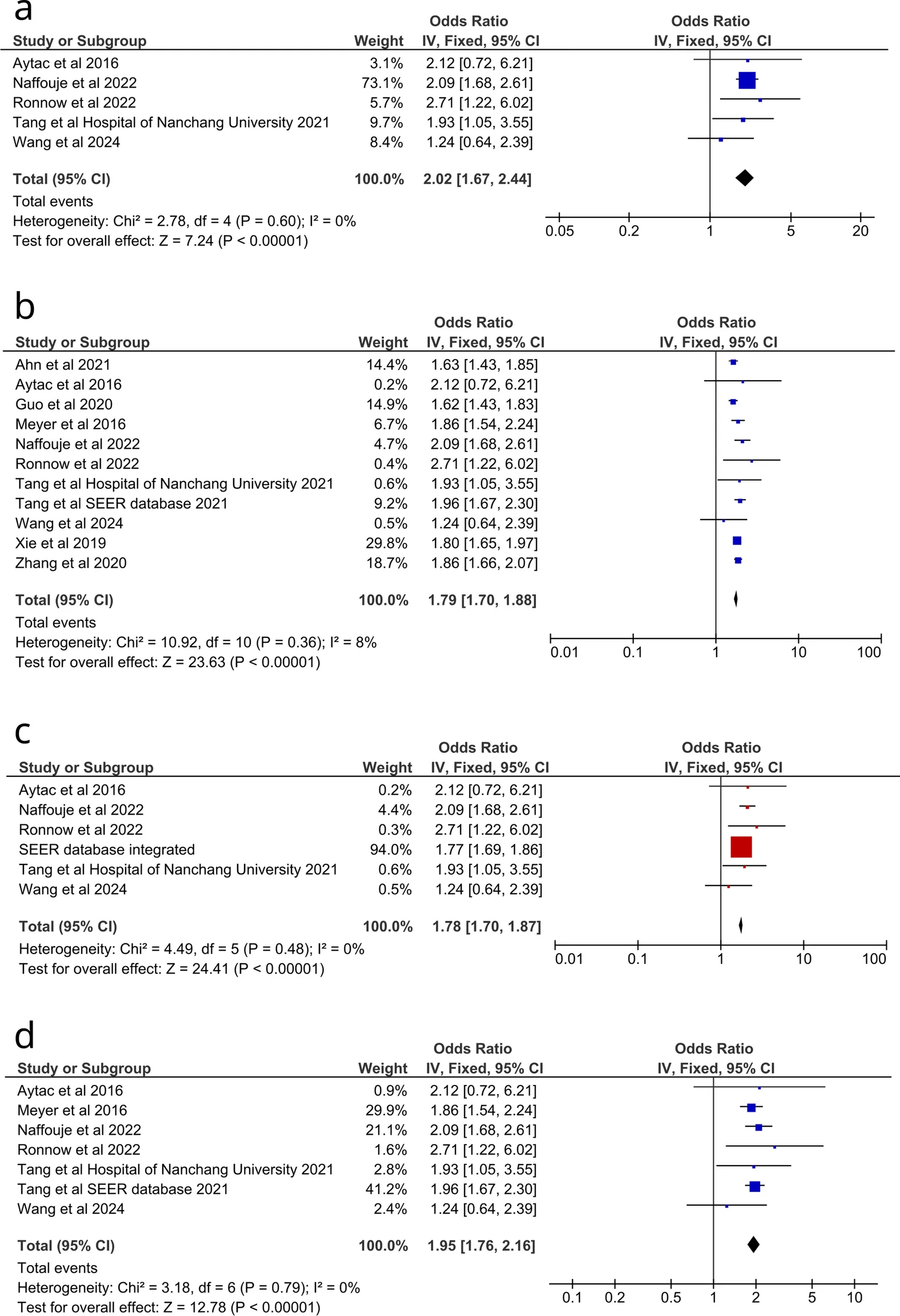
Forest plots with odds ratios of LNM compared between early onset and late-onset T1 CRC in the sensitivity analysis: **a** excluding all SEER data, **b** including all SEER data, **c** integrating SEER data, and **d** including subset data restricted to low-risk LNM. *LNM* lymph-node metastasis, *CRC* colorectal cancer, *SEER* Surveillance Epidemiology and End Results

First, a sensitivity analysis was performed to assess the impact of potential data duplication. Figure 3a shows the sensitivity analysis of five databases in five studies, excluding the SEER data [25–29]. The rate of LNM in EO-T1 CRC remained consistently higher than that in the LO group (a pooled OR: 2.02; 95% CI 1.67–2.44; *p* < 0.05 in the fixed-effect model). Analysis of 11 databases in 10 studies, including all SEER data, also showed a higher LNM rate in EO-T1 CRC (Fig. 3b). In addition, we integrated SEER data into a single dataset and analyzed five other studies [25–29]. The results were similar to the primary analysis (Fig. 3c). Additionally, we conducted a sensitivity analysis including the study by Rönnow et al. [27], which was restricted to patients at low risk for LNM. The results were consistent with those of the primary analysis, with a pooled OR of 1.95 (95% CI 1.76–2.16; *p* < 0.05 in the fixed-effect model; Fig. 3d).

### Evaluation of publication bias

**Fig. 4:**
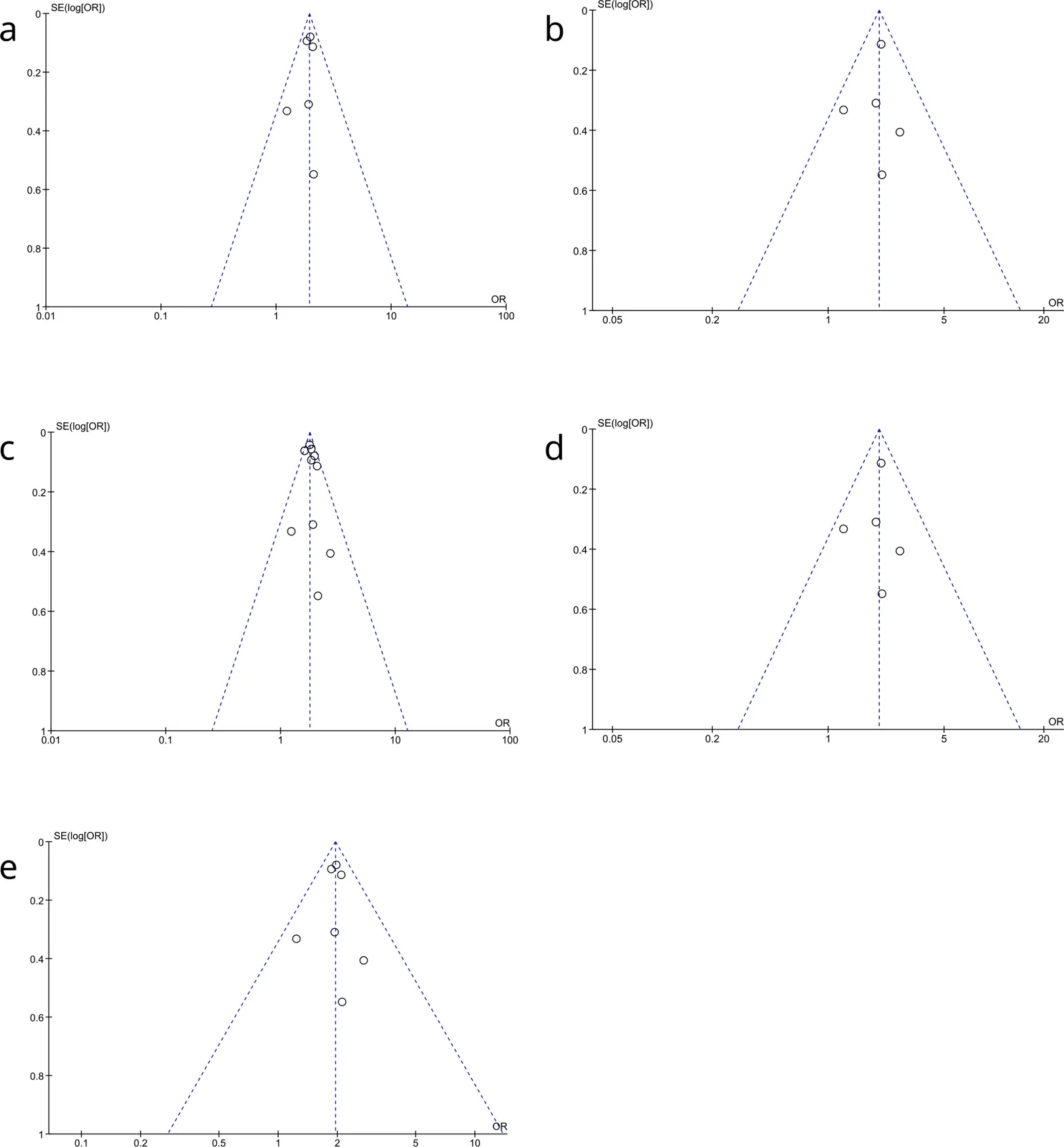
Assessment of publication bias using funnel plots and Egger’s test: **a** funnel plot of the primary analysis, **b** excluding SEER data, **c** including all SEER data, **d** integrated SEER data, and **e** including subset data restricted to low-risk LNM. *SEER* Surveillance, Epidemiology, and End Results

We evaluated the potential publication bias for the primary analysis by constructing funnel plots and performing Egger’s regression analysis (Fig. 4a) and found no apparent asymmetry. Nevertheless, these assessments should be interpreted with caution given the limited number of included studies and the modest statistical power of these methods. Figure 4b, c, d, e shows the evaluation of publication bias for the studies used in each sensitivity analysis using funnel plots. While no clear visual asymmetry was detected, the overlap of data points raises concerns about potential duplication of study populations in analyses involving SEER data.

### Clinicopathological characteristics in EO- and LO-T1 CRC

**Fig. 5:**
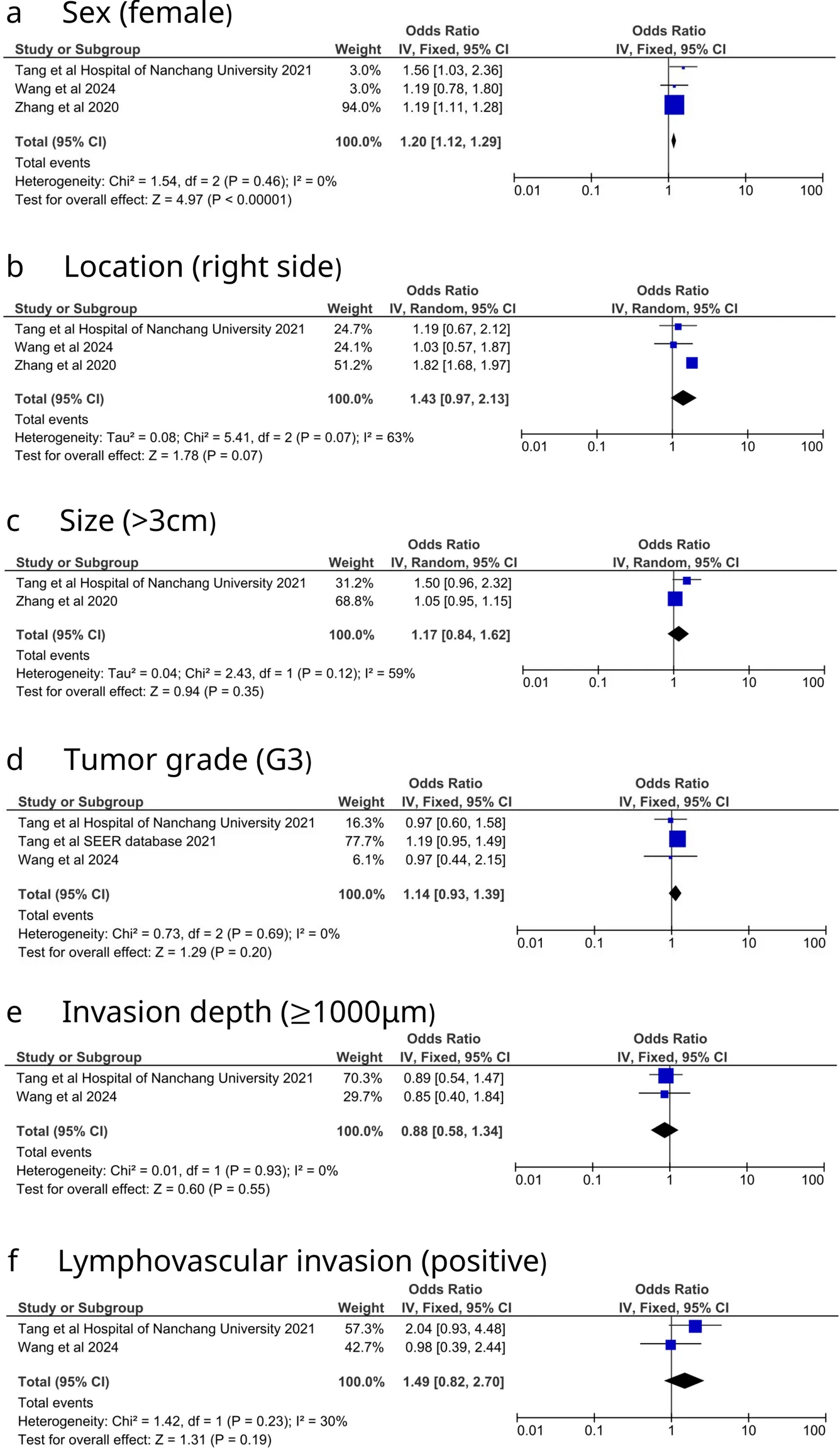
Forest plots for each outcome comparing the odds ratios of clinicopathological characteristics between early onset and late-onset T1 CRC: **a** sex, **b** tumor location, **c** lesion size, **d** tumor grade, **e** invasion depth, and **f** lymphovascular invasion. *CRC* colorectal cancer

The available patient clinicopathological variables were sex, tumor location, size, tumor grade, invasion depth, and lymphovascular invasion status. Table S1 and Fig. 5 summarize the comparisons of clinicopathological features between EO- and LO-T1 CRC. Although the sex distribution in EO-T1 CRC was nearly equal, the proportion of female patients was significantly higher than that in LO-T1 CRC. Left-sided lesions were more frequent than right-sided lesions in both groups, whereas EO-T1 CRC showed a tendency toward a higher proportion of right-sided lesions. No significant differences were observed in tumor grade, size, invasion depth, and lymphovascular invasion status between the two groups.

Furthermore, to assess whether the effect model applied in the analysis was appropriate in the meta-analysis, sensitivity analyses using alternative effect models were performed, which showed consistent results (Fig. S2a–k).

### Adjusted ORs for LNM across different ages

Five databases in four studies conducted multivariable logistic regression analyses that incorporated age < 50 years as a covariate for LNM [17, 26, 28, 33]. Covariates other than age included sex, tumor location, and tumor grade in all studies, and additional covariates incorporated into the analyses were race, lymphovascular invasion, size, invasion depth, lifestyle factors, the number of examined LNs, year of diagnosis, and the status of MLH1, MSH2, EGFR, and ERBB2. However, none of the studies included all of the representative established LNM risk factors such as invasion depth, tumor grade, budding grade, and lymphovascular invasion as covariates; each study lacked at least one of these factors. The covariates included in each study are presented in Table S2.

**Fig. 6:**
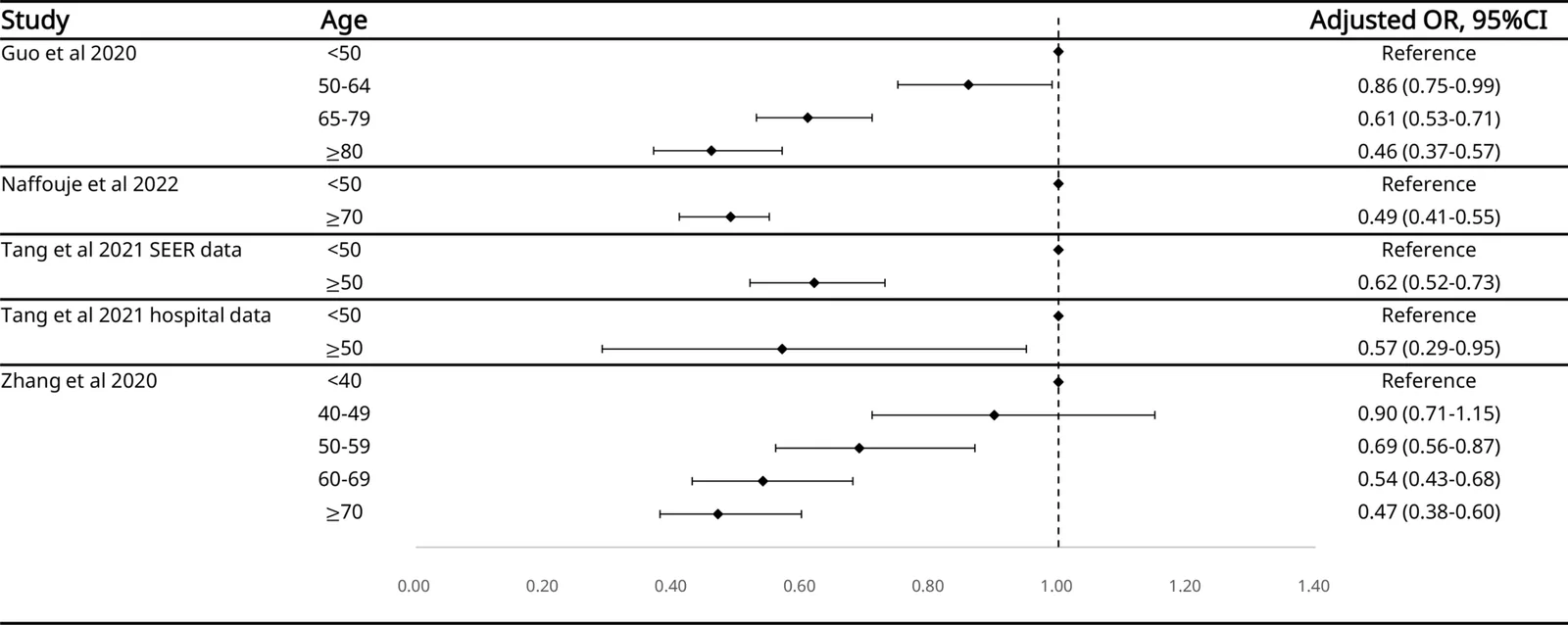
Adjusted odds ratios of LNM across different age groups, calculated from multivariable analyses incorporating age and other factors as covariates. *LNM* lymph-node metastasis, *OR* odds ratio, *CI* confidence interval

Across all studies, younger age tended to be associated with higher ORs for LNM, even after adjustment for these additional factors (Fig. 6). In particular, patients younger than 50 years had a significantly higher odds of LNM than those aged ≥ 80 years, with an adjusted OR of 2.17 (95% CI 1.75–2.70).

## Discussion

The overall rate of LNM in T1 CRC was 11.9% (95% CI 11.5–12.2), which is comparable to those in the previous studies [16–18]. Of these patients, 9.4% were under 50 years of age. The pooled LNM rate was 19.2% (95% CI 17.8–20.6) in EO-T1 CRC and 11.1% (95% CI 10.7–11.5) in LO-T1 CRC, corresponding to an absolute difference of approximately 8%. Compared with LO-T1 CRC, EO-T1 CRC was associated with significantly higher odds of LNM (pooled OR: 1.94; 95% CI 1.75–2.15; p < 0.05 in the fixed-effect model), suggesting a potentially meaningful clinical difference. Although two studies had 95% CIs of the OR crossed one, all six eligible databases showed a trend toward higher LNM rates in EO-T1 CRC, with no significant heterogeneity observed across the studies [25, 29]. To evaluate whether younger age represents an independent risk factor for LNM distinct from these clinicopathological features, it is necessary to assess potential confounding. In the subgroup analysis, the proportion of females was slightly but significantly higher in the EO group. Among individuals aged 20–49 years, the increase in incidence has been reported to be greater for females in several countries [34]. In addition, a previous meta-analysis demonstrated that female sex was associated with a higher risk of LNM in T1 CRC (risk ratio, 2.45; 95% CI 1.03–3.88) [35], suggesting that sex could be related to the higher rate of LNM observed in EO-T1 CRC.

Rectal cancer is also known to be associated with a higher rate of LNM than right-sided colon cancer [36]; however, no significant difference in location was noted between the two groups in this analysis. Furthermore, Meyer et al. [31] had conducted an analysis limited to younger patients with rectal cancer and reported that age was a predictive factor for LNM, even in patients with T1 rectal cancer. Thus, the higher prevalence of rectal cancer in younger patients does not fully explain the higher LNM rate. Additionally, several histopathological features, such as deep submucosal invasion, poor differentiation, lymphovascular invasion, and high-grade tumor budding, are recognized as established risk factors associated with LNM, whereas age has not commonly been regarded as a predictive factor for LNM [19–21]. However, two reports constructed nomograms to predict LNM in T1 CRC using multivariable models, in which age was included as one of the risk factors for LNM [17, 29]. In the present analysis, we conducted a qualitative literature review of studies that calculated adjusted ORs using age and other clinical covariates, including variables generally considered risk factors for LNM, such as tumor location, tumor grade, lymphovascular invasion, tumor size, and invasion depth. Across all four studies [17, 26, 28, 33], even after adjusting for these established risk factors, younger age consistently tended to be associated with higher ORs for LNM. However, because the covariates differed across studies, lymphovascular invasion was incorporated as a covariate in two studies [26, 28] and genetic factors in only one [28]. Thus, although the adjusted ORs were derived from models incorporating multiple covariates, representative risk factors for LNM were not comprehensively included. In addition to established risk factors for LNM, various clinical and social factors, such as healthcare accessibility, income, and treatment selection, may also act as confounders. Therefore, potential confounding factors cannot be fully excluded, and these findings should be interpreted with caution. Accordingly, it cannot be concluded that younger age is an independent risk factor for LNM.

Although evaluation of genetic background is important when investigating EO-CRC, the relationship between genetic factors and LNM remains unclear. Most previous molecular studies have focused on EO-CRC across all stages (hereafter referred to as EO-CRC overall), and investigations specifically targeting EO-T1 CRC remain scarce. Recent reviews have indicated that EO-CRC overall exhibits distinct molecular characteristics compared to LO-CRC, including lower frequencies of KRAS, BRAF, NRAS, and APC mutations; higher frequencies of TP53 and PTEN mutations; and a lower rate of CpG island methylator phenotype positivity [37]. While these molecular characteristics provide important insights, evidence directly linking to the increased risk of LNM in EO-T1 CRC remains limited. Tang et al. [28] investigated molecular characteristics of EO-T1 CRC. However, no significant differences were observed in the gene profiles between younger and older patients. In contrast, a previously reported meta-analysis demonstrated a tendency toward a higher prevalence of MSI in EO-CRC overall (OR: 1.31, 95% CI, 1.11–1.56) [37]. Additionally, one-fifth of EO-CRC overall are considered attributable to hereditary cancer syndromes, with approximately half of these specifically due to Lynch syndrome [38]. High-frequency MSI is suggested to be one of the molecular biological characteristics of EO-CRC overall. However, patients with hereditary CRC, such as Lynch syndrome, or those with high-frequency MSI may have a relatively low likelihood of LNM compared to patients with microsatellite stability (MSS) [39, 40]. Based on these findings, particularly in sporadic and MSS-type EO-CRC, unidentified factors may be associated with LNM risk. Elucidating this unresolved background may contribute to risk stratification, and further molecular and biological investigations are warranted.

The trend toward a higher rate of LNM in younger patients observed in the present analysis may have vital implications for management of EO-T1 CRC. Regarding postoperative surveillance, routine computed tomography (CT) imaging is not mandatory for patients with stage I disease, including T1 CRC [41, 42]. The present meta-analysis evaluated pathological LNM at the time of surgical resection and did not assess postoperative recurrence, distant metastasis, or survival. Therefore, the present findings alone do not justify the routine use of postoperative CT imaging for patients with stage I CRC in clinical practice; however, the higher rate of LNM observed in EO-T1 CRC suggests a potential need for individualized follow-up. Additionally, endoscopic resection is often adopted as primary treatment for T1 CRC; additional surgical resection is recommended in cases with high-risk pathological features, whereas endoscopic management alone is considered acceptable in low-risk cases [42–44]. In the study by Naffouje et al. [26], all patients in both groups underwent endoscopic resection as prior treatment and subsequently underwent additional surgical resection for high-risk lesions; even in this setting, younger age was associated with higher adjusted OR for LNM. However, given the limited number of available studies and the lack of reports evaluating follow-up outcomes in low-risk cases treated with endoscopic resection alone, the present findings cannot be directly applied to patients who underwent endoscopic resection as primary treatment.

Current guidelines do not specifically address the management of EO-T1 CRC; the European Hereditary Tumor Group guideline is the only one that explicitly provides a statement on the management of EO-CRC. This guideline states that insufficient evidence exists to recommend that EO-T1 CRC be managed differently in LO-T1 CRC, indicating that management decisions may not currently be altered solely based on age [45]. However, the higher rate of LNM observed in EO-T1 CRC may indicate a need for more individualized approaches.

Our study has several strengths. To our knowledge, this is the first meta-analysis to specifically focus on age-related differences in LNM in patients with T1 CRC. The large pooled sample size of nearly 30,000 patients, the inclusion of multiple datasets, and consistent findings across the sensitivity and subgroup analyses provided robust evidence supporting the validity of our results.

However, this study had some limitations. This meta-analysis was based on retrospective studies, which were susceptible to various biases. In this context, although a fixed-effect model was used based on low statistical heterogeneity, clinical heterogeneity across studies could not be excluded. Sensitivity analyses using alternative effect models yielded consistent results, supporting the robustness of the findings. The definition of EO-CRC as age < 50 years was established prior to data extraction, as it is the most commonly used cutoff in previous studies. Because the objective of this study was to compare LNM rates between EO- and LO-T1 CRC, including studies with different age definitions could introduce heterogeneity and potential bias. However, the exclusion of studies with different age definitions may itself introduce selection bias. In addition, treatment selection can influence the LNM rate. Importantly, this analysis was restricted to surgically treated patients with pathologically evaluable lymph nodes and did not include patients treated with endoscopic resection alone. Therefore, the observed LNM rates may be overestimated and should be interpreted with caution. They may not be generalizable to the overall T1 CRC population or to low-risk cases.

The assessment of confounding factors also requires thorough interpretation. Although four studies performed multivariable analyses, these results could not be quantitatively integrated into the present meta-analysis, and selection bias and confounding cannot be completely eliminated. Additionally, unpublished data or information that were not reported in the tables or main documents of the included studies could not be incorporated into the analysis. Therefore, the assessment of publication bias is limited by the small number of studies, and the possibility of bias cannot be completely ruled out. Furthermore, information on genetic factors was not available in most studies, preventing molecular biology analyses. Consequently, the present analysis could not fully elucidate the underlying causes of the differences in LNM rates. Further investigations, including molecular biology analyses and large-scale prospective cohort studies, are required.

In conclusion, younger age was associated with higher odds of LNM in surgically treated T1 CRC. Although these findings should be interpreted with caution and may not be generalizable, younger age may represent a candidate risk factor for LNM.

## Supplementary Information

Below is the link to the electronic supplementary material.

Supplementary file1 (DOCX 887 KB)

## Abbreviations

*APC*: Adenomatous polyposis coli
*BRAF*: V-Raf murine sarcoma viral oncogene homolog B
*CRC*: Colorectal cancer
*CT*: Computed tomography
*EGFR*: Epidermal growth factor receptor
*EO-CRC*: Early onset colorectal cancer
*ERBB2*: Erb-B2 receptor tyrosine kinase 2
*LNM*: Lymph-node metastasis
*LO-CRC*: Late-onset colorectal cancer
*MeSH*: Medical Subject Headings
*MLH1*: MutL homolog 1
*MSH2*: MutS homolog 2
*MSI*: Microsatellite instability
*MSS*: Microsatellite stable
*NRAS*: Neuroblastoma rat sarcoma viral oncogene homolog
*PTEN*: Phosphatase and tensin homolog
*SEER*: Surveillance, Epidemiology, and End Results
*TP53*: Tumor protein p53
