# Supplementary material for: Risk of lymph-node metastasis in early onset T1 colorectal cancer: a systematic review and meta-analysis
Source: J Gastroenterol. 2026 Jun 29;61(8):1068–82. doi: 10.1007/s00535-026-02465-7 (PMC13407934; doi:10.1007/s00535-026-02465-7)
Supplement: Supplementary file 1 — Supplementary file1 (DOCX 887 KB) [file 535_2026_2465_MOESM1_ESM.docx]

***Supplementary Information for***

**Risk of Lymph Node Metastasis in Early-Onset T1 Colorectal Cancer: A Systematic Review and Meta-Analysis**

Hiromu Fukuda, MD^1^, Yoshito Hayashi, MD, PhD^1^, Shinji Yoneda, MD^1^, Yujiro Adachi, MD^1^, Ayaka Tajiri, MD^1^, Eiji Kimura, MD, PhD^1^, Ryotaro Uema, MD, PhD^1^, Takeo Yoshihara, MD, PhD^1^, Yoshiki Tsujii, MD, PhD^1^, Takahiro Kodama, MD, PhD^1^, Tetsuo Takehara, MD, PhD^1^

^1^ Department of Gastroenterology and Hepatology, The University of Osaka Graduate School of Medicine

**Correspondence**

Yoshito Hayashi, MD, PhD

Department of Gastroenterology and Hepatology, The University of Osaka Graduate School of Medicine, Yamadaoka2-2, Suita, Osaka 565‑0871, Japan

Tel: (+81) 6-(6879)-3621

E-mail: [y.hayashi@gh.med.osaka-u.ac.jp](mailto:y.hayashi@gh.med.osaka-u.ac.jp)

**Full electronic search methods**

**PubMed/MEDLINE**

(("colorectal neoplasms"[MeSH Terms] OR ("colorectal"[All Fields] AND "neoplasms"[All Fields]) OR "colorectal neoplasms"[All Fields] OR ("colorectal"[All Fields] AND "cancer"[All Fields]) OR "colorectal cancer"[All Fields])) AND ("Neoplasm Invasiveness"[Mesh] OR T1[tiab] OR pT1[tiab] OR pathologicalT1[tiab] OR T1a[tiab] OR pT1a[tiab] OR T1b[tiab] OR pT1b[tiab] OR early[tiab] OR submucosa*[tiab] OR sm[tiab] OR sm1[tiab] OR sm2[tiab] OR sm3[tiab] OR stage1[tiab] OR stageⅠ[tiab] OR superficial invasion[tiab] OR deep submucosal invasion[tiab]) AND (("lymphatic metastasis"[MeSH Terms] OR ("lymphatic"[All Fields] AND "metastasis"[All Fields]) OR "lymphatic metastasis"[All Fields] OR ("lymph"[All Fields] AND "node"[All Fields] AND "metastasis"[All Fields]) OR "lymph node metastasis"[All Fields]　OR “LN metastasis” OR “LNM”)) AND ("young adult"[MeSH Terms] OR “age of onset”[mesh] OR “early-onset”[tiab] OR “early onset”[tiab] OR “EO”[tiab] OR “EO CRC”[tiab] OR “EO-CRC”[tiab] OR “young-onset”[tiab] OR “young onset”[tiab] OR "young adult*" [tiab] OR “younger than 50” OR “50 years”[tiab] or “age 50”[tiab] OR “45 years”[tiab] OR “age 45”[tiab])

**SCOPUS**

(("colorectal neoplasms" OR ("colorectal" AND "neoplasms") OR "colorectal cancer" OR ("colorectal" AND "cancer"))) AND TITLE-ABS-KEY(("Neoplasm Invasiveness" OR T1 OR pT1 OR pathologicalT1 OR T1a OR pT1a OR T1b OR pT1b OR early OR submucosa* OR sm OR sm1 OR sm2 OR sm3 OR stage1 OR "stage I" OR "superficial invasion" OR "deep submucosal invasion")) AND TITLE-ABS-KEY(("lymphatic metastasis" OR ("lymphatic" AND "metastasis") OR "lymph node metastasis" OR "LN metastasis" OR "LNM")) AND TITLE-ABS-KEY(("young adult" OR "age of onset" OR "early-onset" OR "early onset" OR "EO" OR "EO CRC" OR "EO-CRC" OR "young-onset" OR "young onset" OR "young adult*" OR "younger than 50" OR "50 years" OR "age 50" OR "45 years" OR "age 45"))

**Web of Science**

TS=("colorectal neoplasms" OR ("colorectal" AND "neoplasms") OR "colorectal cancer" OR ("colorectal" AND "cancer"))

AND TS=("Neoplasm Invasiveness" OR “T1” OR “pT1” OR “pathologicalT1” OR “T1a” OR “pT1a” OR “T1b” OR “pT1b” OR “early” OR “submucosa*” OR “sm” OR “sm1” OR “sm2” OR “sm3” OR “stage1” OR "stage I" OR "superficial invasion" OR "deep submucosal invasion")

AND TS=("lymphatic metastasis" OR ("lymphatic" AND "metastasis") OR "lymph node metastasis" OR "LN metastasis" OR "LNM")

AND TS=("young adult" OR "age of onset" OR "early-onset" OR "early onset" OR EO OR "EO CRC" OR "EO-CRC" OR "young-onset" OR "young onset" OR "young adult*" OR "younger than 50" OR "50 years" OR "age 50" OR "45 years" OR "age 45")

**Cochrane Library**


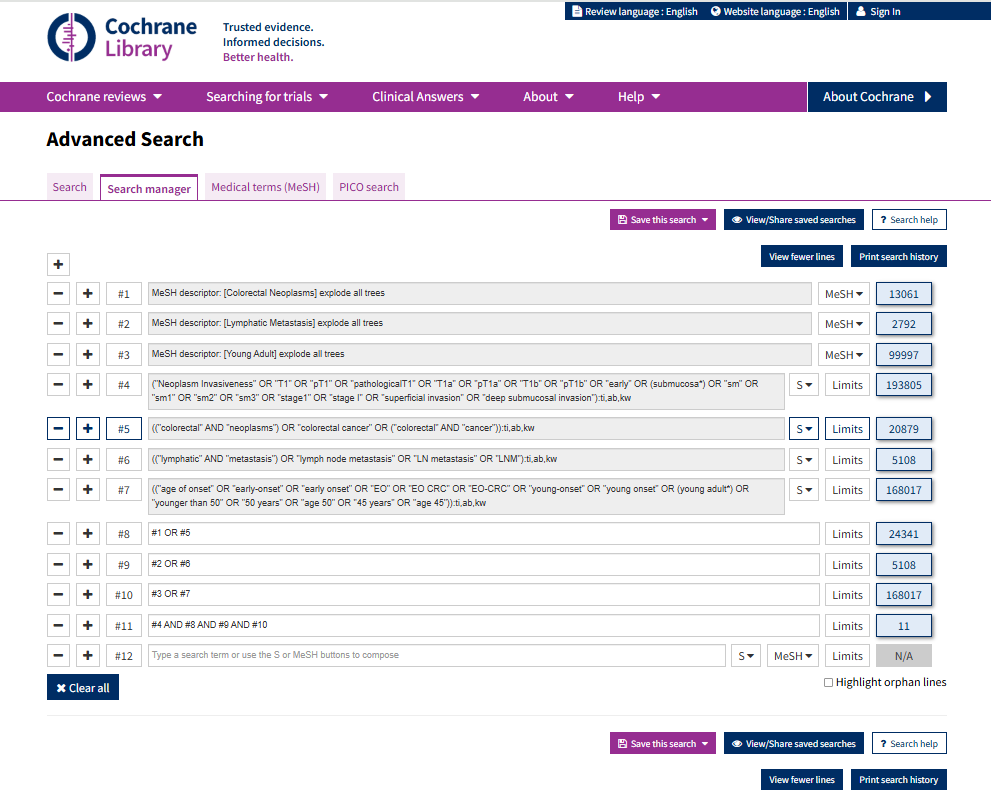


**ICHUSHI**

(((大腸腫瘍/TH or 大腸がん/AL) or (大腸腫瘍/TH or 大腸癌/AL) or (大腸腫瘍/TH or 大腸腫瘍/AL) or 大腸悪性腫瘍/AL or colorectal/AL and (腫瘍/TH or cancer/AL) or colorectal/AL and (腫瘍/TH or neoplasms/AL)) and (T1/AL or pT1/AL or 病理学的T1/AL or T1a/AL or pT1a/AL or T1b/AL or pT1b/AL or 粘膜下浸潤/AL or submucosa/AL or 粘膜下層浸潤/AL or 深部粘膜下浸潤/AL or stage1/AL or ステージ1/AL or superficial/AL and invasion/AL or deep/AL and submucosal/AL and invasion/AL) and ((リンパ行性転移/TH or リンパ節転移/AL) or リンパ節転移率/AL or (リンパ液/TH or lymph/AL) and node/AL and (腫瘍転移/TH or metastasis/AL) or lymphatic/AL and (腫瘍転移/TH or metastasis/AL) or LN/AL and (腫瘍転移/TH or metastasis/AL) or LNM/AL) and (若年/AL or 若年成人/AL or 若年発症/AL or 早期発症/AL or young/AL and (成人/TH or adult/AL) or early-onset/AL or young-onset/AL or 50歳未満/AL or 50歳以下/AL or 45歳未満/AL or 45歳以下/AL or 若い患者/AL))

Hand Search

reference

1. Tang CT, Guo ZX, Wang P, Chen YX, Zeng CY. Higher LNM rate and poorer prognosis of early-onset compared to late-onset T1 stage colorectal cancer: a large-population based study. Am J Cancer Res. 2021 Jun 15;11(6):3176-3188. PMID: 34249453; PMCID: PMC8263633.
2. Ramai D, Singh J, Facciorusso A, Chandan S, Dhindsa B, Dhaliwal A, Galassi B, Tomasello G, Ghidini M. Predictors of Lymph Node Metastasis in T1 Colorectal Cancer in Young Patients: Results from a National Cancer Registry. J Clin Med. 2021 Nov 25;10(23):5511. doi: 10.3390/jcm10235511. PMID: 34884212; PMCID: PMC8658610.
3. Wang K, He H, Lin Y, Zhang Y, Chen J, Hu J, He X. A new clinical model for predicting lymph node metastasis in T1 colorectal cancer. Int J Colorectal Dis. 2024 Apr 3;39(1):46. doi: 10.1007/s00384-024-04621-y. PMID: 38565736; PMCID: PMC10987358.
4. Meyer JE, Cohen SJ, Ruth KJ, Sigurdson ER, Hall MJ. Young Age Increases Risk of Lymph Node Positivity in Early-Stage Rectal Cancer. J Natl Cancer Inst. 2016 Jan;108(1):djv284. doi: 10.1093/jnci/djv284. PMID: 26719881; PMCID: PMC4715232.
5. Guo K, Feng Y, Yuan L, Wasan HS, Sun L, Shen M, Ruan S. Risk factors and predictors of lymph nodes metastasis and distant metastasis in newly diagnosed T1 colorectal cancer. Cancer Med. 2020 Jul;9(14):5095-5113. doi: 10.1002/cam4.3114. Epub 2020 May 29. PMID: 32469151; PMCID: PMC7367623.
6. Wang H, Lu H, Yang H, Zhang X, Thompson EW, Roberts MS, Hu Z, Liang X, Li X. Impact of Age on Risk of Lymph Node Positivity in Patients with Colon Cancer. J Cancer. 2019 May 12;10(9):2102-2108. doi: 10.7150/jca.28377. PMID: 31205571; PMCID: PMC6548175.

| Table S1 Clinicopathological characteristics of early-onset and late-onset T1 CRC | | | | | |
| --- | --- | --- | --- | --- | --- |
| Characteristics | Study | early-onset T1 CRC | | late-onset T1 CRC | |
| Sex |  | female | male | female | male |
|  | Tang et al 2021 hospital data [28] | 60 (50.4%) | 59 (49.6%) | 141 (39.5%) | 216 (60.5%) |
|  | Wang et al 2024 [29] | 53 (48.2%) | 57 (51.8%) | 205 (43.9%) | 262 (56.1%) |
|  | Zhang et al 2020 [33] | 1624 (53.2%) | 1428 (46.8%) | 15360 (48.8%) | 16094 (51.2%) |
| Location |  | left | right | left | right |
|  | Tang et al 2021 hospital data [28] | 100 (84.0%) | 19 (16.0%) | 308 (86.3%) | 49 (13.7%) |
|  | Wang et al 2024 [29] | 94 (85.4%) | 16 (14.6%) | 401 (85.9%) | 66 (14.1%) |
|  | Zhang et al 2020 [33] | 1983 (65.0%) | 1069 (35.0%) | 24274 (77.1%) | 7180 (22.9%) |
| Size |  | ≤3cm | >3cm | ≤3cm | >3cm |
|  | Tang et al 2021 hospital data [28] | 76 (69.1%) | 43 (30.9%) | 259 (72.5%) | 98 (27.5%) |
|  | Zhang et al 2020 [33] | 2434 (79.7%) | 618 (20.3%) | 25308 (80.5%) | 6146 (19.5%) |
| Tumor grade |  | G1 or G2 | G3 | G1 or G2 | G3 |
|  | Tang et al 2021 hospital data [28] | 91 (76.5%) | 28 (23.5%) | 271 (75.9%) | 86 (24.1%) |
|  | Tang et al 2021 SEER data [28] | 947 (91.1%) | 93 (8.9%) | 11127 (92.4%) | 917 (7.6%) |
|  | Wang et al 2024 [29] | 102 (92.7%) | 8 (7.3%) | 432 (92.5%) | 35 (7.5%) |
| Invasion depth |  | <1000μm | ≥1000μm | <1000μm | ≥1000μm |
|  | Tang et al 2021 hospital data [28] | 27 (22.7%) | 92 (77.3%) | 74 (20.7%) | 283 (79.3%) |
|  | Wang et al 2024 [29] | 9 (8.2%) | 101 (91.8%) | 33 (7.1%) | 434 (92.9%) |
| Lymphovascular invasion | | positive | negative | positive | negative |
|  | Tang et al 2021 hospital data [28] | 11 (9.2%) | 108 (90.8%) | 17 (4.8%) | 340 (95.2%) |
|  | Wang et al 2024 [29] | 6 (5.5%) | 104 (94.5%) | 26 (5.6%) | 441 (94.4%) |

*T1 CRC* T1 colorectal cancer, *EO* early-onset, *LO* late-onset, *G1* well-differentiated, *G2* moderately differentiated, *G3* poorly differentiated, *SEER* Surveillance, Epidemiology, and End Results

| **Table S2 Logistic regression analysis of risk factors for lymph node metastasis with young age included as a covariate** | | | | | | | | |
| --- | --- | --- | --- | --- | --- | --- | --- | --- |
| **Study** | **Age** | **Adjusted OR** | **95%CI** | **P value** | **Covariate other than age** | | | |
| **Guo et al 2020 [17]** | <50 | reference |  |  | Sex | Location | | Tumor grade |
|  | 50-64 | 0.86 | 0.75-0.99 | 0.36 | Race | Size | | Examined LNs |
|  | 65-79 | 0.61 | 0.53-0.71 | <0.001 | Histology | Year of diagnosis | | Marital status |
|  | >80 | 0.46 | 0.37-0.57 | <0.001 | Regional nodes examined | |  | |
| **Naffouje et al 2022 [26]** | <50 | reference |  |  | Sex | Location | | Tumor grade |
|  | ＞70 | 0.5 | 0.41-0.55 | <0.001 | Lymphovascular invasion |  | |  |
| **Tang et al 2021 SEER data**  **[28]** | <50 | reference |  |  | Sex | Location | | Tumor grade |
|  | >50 | 0.62 | 0.53-0.73 | <0.001 | Race | Size | | Examined LNs |
| **Tang et al 2021 hospital data [28]** |  |  |  |  | Sex | Location | | Tumor grade |
|  | <50 | reference |  |  | Race | Size | | Examined LNs |
|  | >50 | 0.57 | 0.29-0.95 | 0.042 | Lymphovascular invasion | Invasion depth | | Smoking and Drinking |
|  |  |  |  |  | MLH1, MSH2, EGFR, ERRB2 status | | | |

| **Zhang et al 2020 [33]** | <40 | reference |  |  | Sex | Location | Tumor grade |
| --- | --- | --- | --- | --- | --- | --- | --- |
|  | 40-49 | 0.9 | 0.71-1.15 | 0.37 | Race | Size | Examined LNs |
|  | 50-59 | 0.69 | 0.56-0.87 | 0.001 | Year of diagnosis | T stage |  |
|  | 60-69 | 0.54 | 0.43-0.68 | <0.001 |  |  |  |

*OR* odds ratio, *CI* confidence interval, *LN* lymph node, *SEER* Surveillance Epidemiology and End Results, *MLH1* MutL homolog 1, *MSH2* MutS homolog 2, *EGFR* epidermal growth factor receptor, *c-erbB1* erythroblastic leukemia viral oncogene homolog 1

**
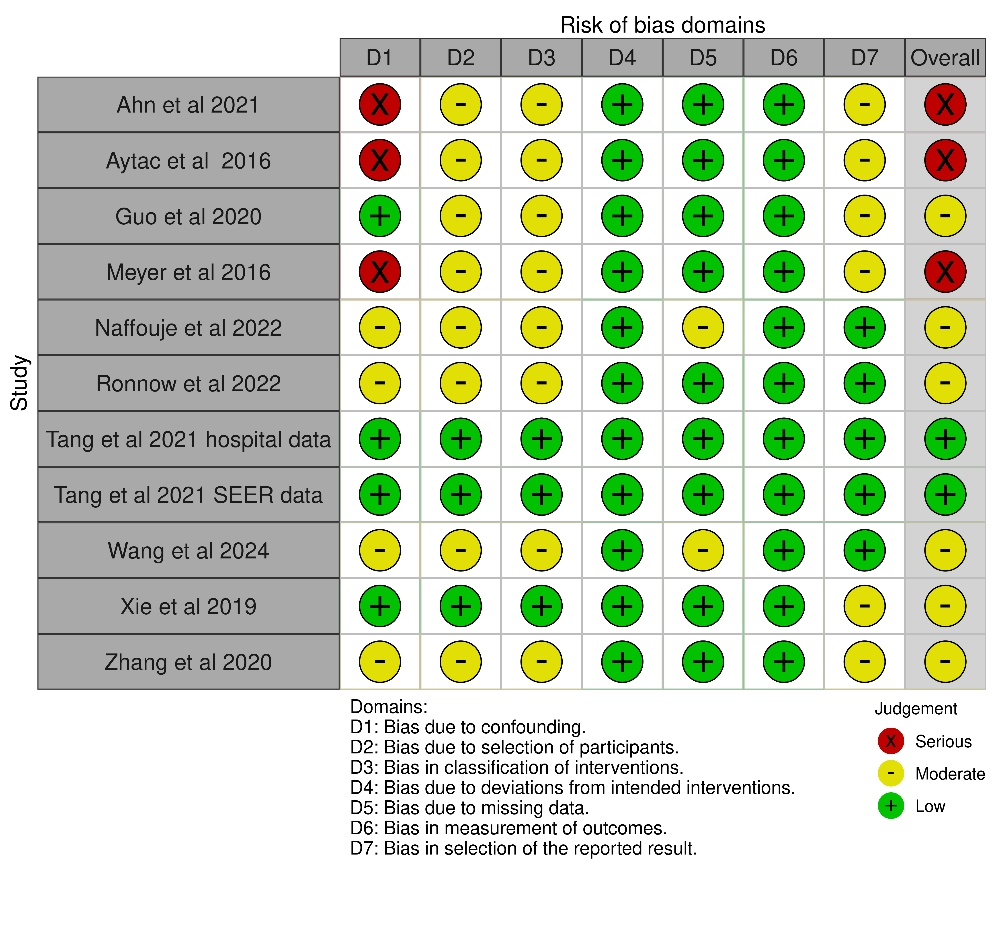
**

**Fig. S1** Risk of bias assessment for individual studies according to the risk of bias in nonrandomized studies of interventions (ROBINS-I) tool version 2.

*SEER* Surveillance Epidemiology and End Results

**
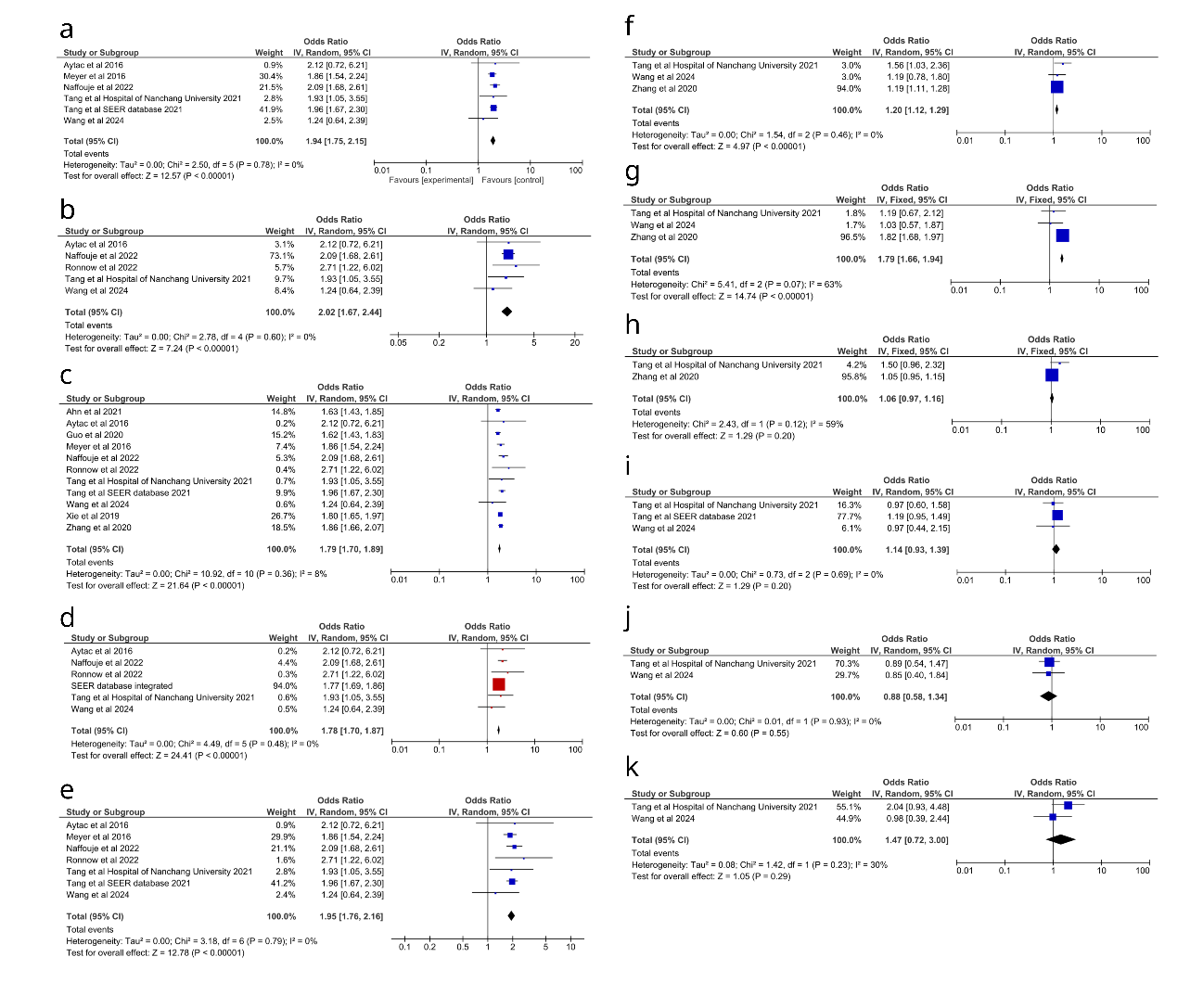
**

**Fig. S2 a–k** Sensitivity analyses using alternative models to assess the robustness of the findings. Random-effects models were applied to outcomes without significant heterogeneity and fixed-effects models to those with heterogeneity. The results were consistent between the fixed- and random-effects models. **a** Primary analysis (random-effects), **b** excluding all SEER data (random-effects), **c** including all SEER data (random-effects), **d** integrating SEER data (random-effects), **e** including subset data restricted to low-risk LNM (random-effects), **f** sex (random-effects), **g** location (fixed-effects), **h** lesion size (fixed-effects), **i** tumor grade (random-effects), **j** invasion depth (random-effects), and **k** lymphovascular invasion (random-effects).

*OR* odds ratio, *CI* confidence interval, *SEER* Surveillance, Epidemiology, and End Results
